# Epidemiology, prognostic factors, and survival analysis in small cell esophageal carcinoma: A population-based study with external validation

**DOI:** 10.17305/bb.2024.11090

**Published:** 2024-09-03

**Authors:** Jiahao Zhu, Benjie Xu, Yuanyuan Li, Xiangyi Pang, Shengjun Ji, Jie Lian, Haibo Lu

**Affiliations:** 1 Department of Outpatient Chemotherapy, Harbin Medical University Cancer Hospital, Harbin, China; 2 Department of Radiotherapy and Oncology, The Affiliated Suzhou Hospital of Nanjing Medical University, Gusu School, Nanjing Medical University, Suzhou, China

**Keywords:** Esophageal carcinoma, small cell carcinoma, epidemiology, prognosis, treatment

## Abstract

Small cell esophageal carcinoma (SCEC) is a poorly differentiated esophageal neuroendocrine neoplasm with a poor prognosis. This study aimed to explore the factors and treatment approaches influencing the prognosis of SCEC. In this retrospective study, we collected data from the Surveillance, Epidemiology, and End Results (SEER) 18 registries cohort between 2004 and 2019, as well as from a Chinese institutional registry covering the period from 2012 to 2022. We assessed the annual percentage change (APC) in incidence of SCEC. Kaplan–Meier and Cox regression analyses were conducted to evaluate survival outcomes. Additionally, nomograms were developed for overall survival (OS) and cancer-specific survival (CSS) in the SEER cohort for SCEC and validated in an independent Chinese cohort. This analysis included 299 SCEC patients from the SEER cohort and 66 cases from the Chinese cohort. During the period of 2004–2019, the incidence of SCEC reached a plateau, with an APC of −1.40 (95% confidence interval [CI]: −4.3 to 1.40, *P* > 0.05). Multivariable Cox regression analysis revealed that age, distant metastasis, and chemotherapy were independent factors for OS, while distant metastasis and chemotherapy were independent factors for CSS. The nomograms developed for OS and CSS in SCEC exhibited remarkable accuracy and reliable predictive capacity in estimating 1-year, 3-year, and 5-year OS and CSS. SCEC is a rare malignancy with aggressive behavior. Distant metastasis is significantly associated with worse OS and CSS in patients with SCEC. Currently, chemotherapy remains the primary treatment approach for SCEC.

## Introduction

Primary small cell esophageal carcinoma (SCEC) is a rare histological variant, accounting for approximately 0.5%–3.8% of all esophageal malignant neoplasms [1, 2]. This subtype is characterized by its aggressive nature and a tendency to metastasize to distant organs and lymph nodes [3]. According to the updated classification of digestive system neuroendocrine neoplasms by the World Health Organization in 2019, both SCEC and large cell esophageal carcinoma (LCEC) are classified as poorly differentiated neuroendocrine carcinomas (NECs) [4]. Most esophageal NECs are of the small-cell type. Histologically, SCEC is characterized by small cancer cells of uniform size, arranged in a linear pattern with indistinct boundaries [5]. In contrast, LCEC displays tumor cells that are more than three times larger than lymphocytes [6]. An accurate diagnosis can be made by combining cytological characteristics, immunohistochemistry, and positron emission tomography scans. Unfortunately, the prognosis for SCEC is generally unfavorable, and no optimal treatment approach has been established for this condition. Current management strategies for SCEC are based on treatment guidelines for small-cell lung cancer, typically involving platinum-based combination therapy [7, 8]. There is also no consensus on the optimal second-line therapy for SCEC. Due to the low incidence of SCEC, no prospective studies have been conducted on its treatment. Therefore, retrospective studies are valuable in providing insights into SCEC. By analyzing historical data, these studies can offer unique insights into the disease’s epidemiology, risk factors, and prognostic indicators, thereby bridging critical knowledge gaps and informing future research and clinical practice. This study aimed to investigate incidence trends among SCEC patients, identify independent prognostic factors using data from the Surveillance, Epidemiology, and End Results (SEER) cancer registry, and elucidate potentially effective therapies. Additionally, we developed a prognostic nomogram based on risk factors associated with SCEC-related mortality, offering valuable prognostic information for both patients and clinicians.

## Materials and methods

### Patients’ collection

Clinical and survival data on patients diagnosed with SCEC between 2004 and 2019, classified according to the International Classification of Diseases (ICD), were obtained from the SEER 18 Custom Data. The extraction process was performed using SEER*Stat version 8.4.0, which included additional treatment fields. Patients with SCEC had to meet the following criteria: (1) ICD for oncology 3rd edition codes (SCEC: 8041/3, 8042/3, 8043/3, 8044/3, and 8045/3); (2) topographical codes: C15.0–C15.5, C15.8, and C15.9 (Table S1); and (3) clinicopathological parameters, including gender, age, race, tumor location, TNM stage (based on the 7th edition criteria of the American Joint Committee on Cancer), metastasis site, therapeutic methods, and survival data. Patients with more than one primary tumor or a survival time of zero were excluded from the study. Data from patients diagnosed with SCEC between February 2012 and August 2022 in China were collected for nomogram validation. This study’s data review was conducted with institutional review board approval. All participating patients provided informed consent. This study complied with the Declaration of Helsinki.

### Endpoint definition

The endpoints of this study were defined as follows: Overall survival (OS) represents the interval from the primary diagnosis of SCEC to death or the last recorded visit; Cancer-specific survival (CSS) represents the interval between the initial diagnosis of the disease and death related to SCEC.

### Epidemiological analysis

The incidence rates of SCEC were calculated and adjusted for age based on the 2000 US population, representing the number of new occurrences per 1,000,000 person-years. The annual percentage change (APC) was determined using the weighted least squares method. To analyze the incidence trend, the percentage change was compared to zero.

### Nomogram construction for OS and CSS and validation

Univariate and multivariate Cox regression analyses were conducted for OS and CSS. The proportional hazard hypothesis test was used to evaluate prognostic factors. Factors with *P* values below 0.05 in the univariate Cox analysis were incorporated into the multivariate Cox regression model to identify independent prognostic indicators. Subsequently, nomograms were constructed based on the results of the multivariate Cox analysis in the training cohort. These nomograms integrated all independent prognostic factors and served as predictive tools for estimating the risk of patients surviving less than one, three, or five years. Each variable in the nomogram was assigned a specific number of points along a horizontal axis. By summing the points corresponding to each patient’s variables, a risk score was obtained. The nomogram provided estimates for 1-, 3-, and 5-year OS and CSS rates based on the calculated risk score. To further stratify patients based on predicted prognosis, X-tile software (version 3.6.1) was used. The individual risk score for each patient in the training cohort was computed using the nomogram. Patients were then classified into low-risk, medium-risk, or high-risk groups based on their respective risk scores. This categorization allowed for additional stratification of patients according to their predicted prognosis. We assessed the predictive performance of the nomogram model for OS and CSS in the training and validation cohorts using calibration curves and decision curve analysis (DCA). We evaluated the accuracy of the nomogram model through the examination of receiver operating characteristic (ROC) curves and the time-dependent concordance index (C-index).

### Ethical statement

The authors state that approval was obtained from the institutional review board of The Harbin Medical University Cancer Hospital, and that all human experimental investigations followed the principles outlined in the Declaration of Helsinki. Additionally, informed consent was obtained from all participants involved in the study.

### Statistical analysis

The chi-square test or Fisher’s exact test was used to compare characteristics among different groups. For continuous variables, the Student’s *t*-test and Wilcoxon’s rank-sum test were performed. Kaplan–Meier curves were drawn for OS and CSS, and the differences were evaluated using the log-rank test. A two-sided *P* < 0.05 was considered statistically significant. R software version 4.2.0 was used for all statistical analyses. The R packages used in the analysis included “survival,” “survminer,” “rms,” “regplot,” “ggDCA”, and “pec.”

## Results

### Epidemiology trends and patient characteristics

**Figure 1.**
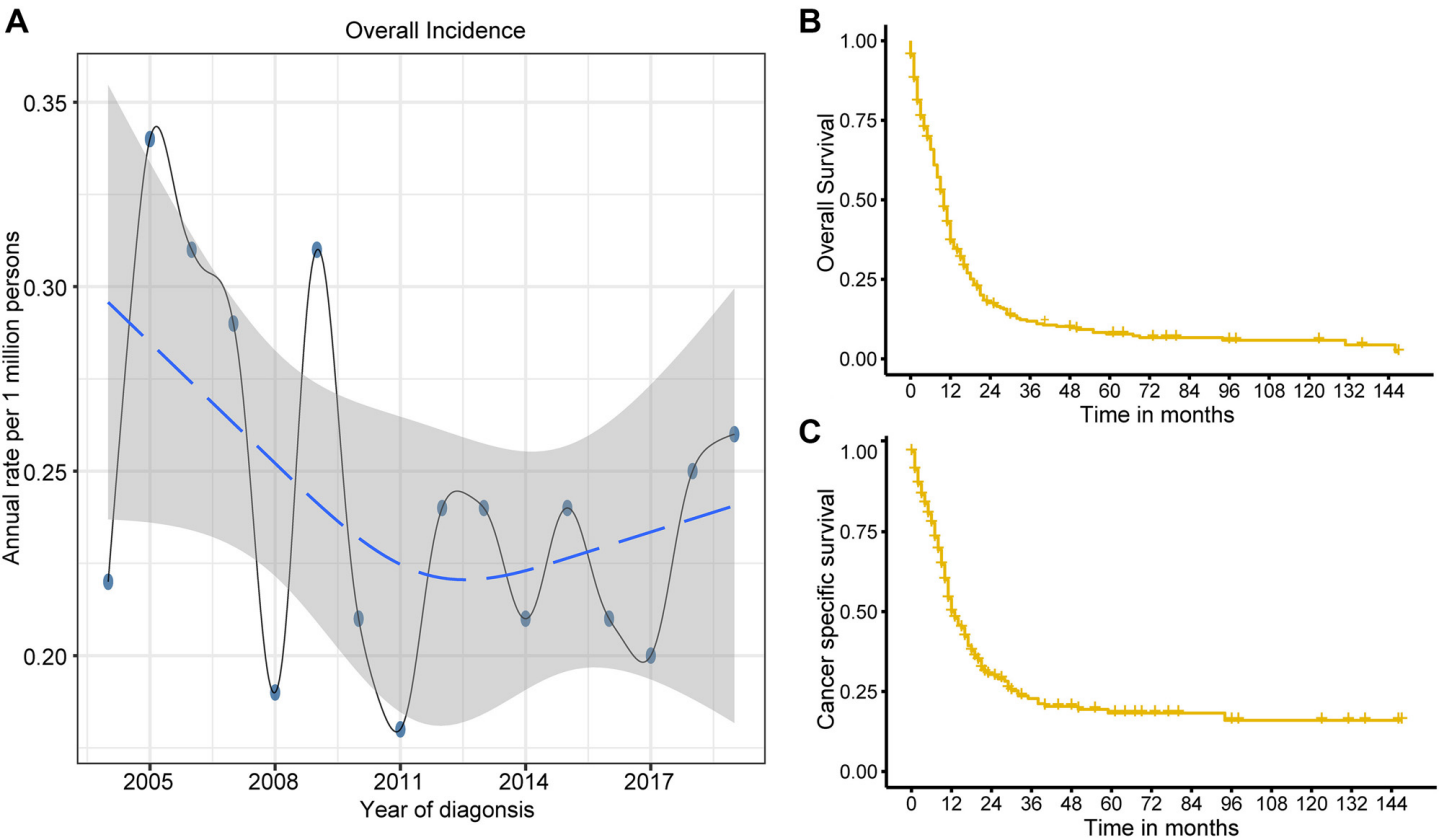
**Epidemiology trends and survival analysis of SCEC.** The annual age-adjusted incidence of SCEC (A) from 2004 to 2019. Kaplan–Meier survival curves for OS (B) and CSS (C) in SCEC from 2004 to 2019. SCEC: Small cell esophageal carcinoma; OS: Overall survival; CSS: Cancer-specific survival.

Due to limitations in population data acquisition, the incidence rates of SCEC between 2004 and 2019 were calculated. The overall age-adjusted incidence of SCEC was 0.213 per 1,000,000 per year during this period. The APC for SCEC was --1.40 (95% confidence interval [CI]: --4.3 to 1.40, *P* > 0.05). The overall incidence of SCEC has plateaued over the last 15 years (Figure 1A).

This study included 299 SCEC patients from the SEER cohort (2004–2019) and 66 cases from the Chinese cohort (2012–2022) for survival analysis. Among the SEER cohort, males accounted for 68.6% and females for 31.4%. In contrast, most patients in the Chinese cohort were male (93.9%). The proportion of individuals ≤ 65 years old was 40.8%, similar to the 45.5% observed in the Chinese population. Both cohorts showed that the middle and lower thirds of the esophagus were the most common locations of SCEC. Approximately half of the patients had distant metastasis at initial diagnosis in the SEER cohort (46.8%), with a higher rate observed in the Chinese cohort (75.8%). More than half of the patients in both datasets had lymph node metastasis and tumor sizes exceeding 20 mm. Among the 44 (66.7%) patients who received chemotherapy, 32 were treated with the Etoposide and Cisplatin regimen, while 12 received the Irinotecan and Cisplatin regimen. A summary of the clinical characteristics of SCEC in this study is shown in Table 1.

**Table 1:** Characteristics of SCEC patients enrolled in this study

**Characteristics**	**SEER SCEC cohort**	**Chinese SCEC cohort**
	***N* ═ 299**	***N* ═ 66**
*Gender*		
Male	205 (68.6%)	62 (93.9%)
Female	94 (31.4%)	4 (6.1%)
*Age at diagnosis, years*		
≤ 65	122 (40.8%)	30 (45.5%)
> 65	177 (59.2%)	36 (54.5%)
*Race*		
White	233 (77.9%)	0 (0%)
Black	33 (11%)	0 (0%)
Asian/PI/AI	33 (11%)	66 (100%)
*Tumor location*		
Upper	21 (7%)	5 (7.6%)
Middle	75 (25.1%)	35 (53.0%)
Lower	165 (55.2%)	26 (39.4%)
Unknown	38 (12.7%)	0 (0%)
*T category*		
T1-2	87 (29.1%)	34 (51.5%)
T3-4	109 (36.5%)	32 (48.5%)
Unknown	103 (34.4%)	0 (0%)
*Lymph node metastasis*		
Negative	108 (36.1%)	23 (34.8%)
Positive	152 (50.8%)	43 (65.2%)
Unknown	39 (13%)	0 (0%)
*Distant metastasis*		
Yes	145 (48.5%)	50 (75.8%)
No	154 (51.5%)	16 (24.2%)
*TNM stage*		
I-II	66 (22.1%)	15 (22.7%)
III-IV	192 (64.2%)	51 (77.3%)
Unknown	41 (13.7%)	0 (0%)
*SEER stage*		
Regional	72 (24.1%)	11 (16.7%)
Localized	49 (16.4%)	5 (7.5%)
Distant	178 (59.5%)	50 (75.8%)
*Tumor size, mm*		
≤ 20	23 (7.7%)	24 (36.4%)
> 20	158 (52.8%)	42 (63.6%)
Unknown	118 (39.5%)	0 (0%)
*Bone metastasis*		
No	163 (54.5%)	62 (93.9%)
Yes	19 (6.4%)	4 (6.1%)
Unknown	117 (39.1%)	0 (0%)
*Brain metastasis*		
No	178 (59.5%)	62 (93.9%)
Yes	4 (1.3%)	4 (6.1%)
Unknown	117 (39.1%)	0 (0%)
*Liver metastasis*		
No	117 (39.1%)	58 (87.9%)
Yes	64 (21.4%)	8 (12.1%)
Unknown	118 (39.5%)	0 (0%)
*Lung metastasis*		
No	160 (53.5%)	64 (97.0%)
Yes	20 (6.7%)	2 (3.0%)
Unknown	119 (39.8%)	0 (0%)
*Surgery*		
No	278 (93%)	32 (48.5%)
Yes	21 (7%)	34 (51.5%)
*Radiotherapy*		
No	158 (52.8%)	25 (37.9%)
Yes	141 (47.2%)	41 (62.1%)
*Chemotherapy*		
No	75 (25.1%)	22 (33.3%)
Yes	224 (74.9%)	44 (66.7%)

SEER: Surveillance, Epidemiology, and End Results; SCEC: Small cell esophageal carcinoma.

### Prognostic factors and survival analysis

The median OS and CSS were 10 months (95% CI 9–11) and 12 months (95% CI 11–16), respectively, for SCEC patients. The 1-, 3-, and 5-year OS rates were 36.9%, 11.9%, and 7.8%, respectively, while the CSS rates were 49.9%, 22.8%, and 18.3%, respectively (Figure 1B and 1C).

**Figure 2.**
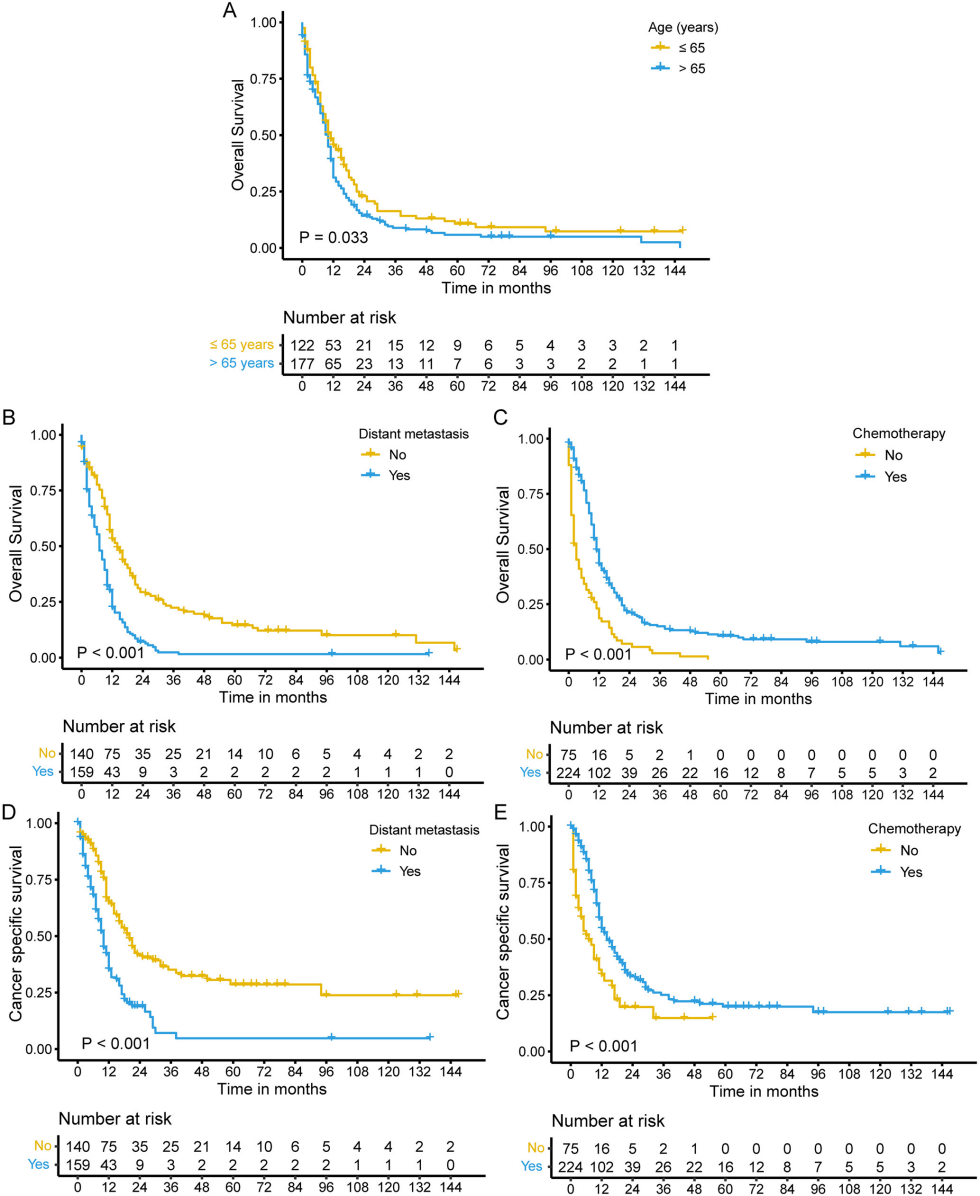
**Effect of age, distant metastasis, and chemotherapy on the prognosis of SCEC patients in the SEER cohort.** Kaplan–Meier survival curves for OS in SCEC patients aged ≤ 65 years and > 65 years (A), with and without distant metastasis (B), and with and without chemotherapy (C). Kaplan–Meier survival curves for CSS in SCEC patients with and without distant metastasis (D) and with and without chemotherapy (E). SCEC: Small cell esophageal carcinoma; OS: Overall survival; CSS: Cancer-specific survival; SEER: Surveillance, Epidemiology, and End Results.

Univariate and multivariate Cox regression analyses were conducted to determine independent prognostic factors for OS and CSS in the SEER cohort. After univariate analysis for OS, variables with a *P* < 0.05, including age, distant metastasis, surgery, radiation therapy, and chemotherapy, were included in the multivariate Cox analysis. Age (HR ═ 1.31; 95% CI ═ 1.01–1.69; *P* ═ 0.039), distant metastasis (HR ═ 2.04; 95% CI ═ 1.49–3.80; *P* < 0.001), and chemotherapy (HR ═ 0.40; 95% CI ═ 0.30–0.54; *P* ═ 0.001) were identified as independent factors for OS (Table 2). SCEC patients younger than 65 years, without distant metastasis, and receiving chemotherapy had better OS (Figure 2A–2C). In multivariate analysis for CSS, distant metastasis (HR ═ 2.17; 95% CI ═ 1.47–3.19; *P* < 0.001) and chemotherapy (HR ═ 0.45; 95% CI ═ 0.31–0.65; *P* < 0.001) were identified as independent factors for CSS (Table 2). SCEC patients without distant metastasis and those receiving chemotherapy had prolonged CSS (Figure 2D and 2E).

**Table 2:** Univariate and multivariate analysis for OS and cancer specific survival in SCEC patients

**Variables**	**UVA (OS)**			**MVA (OS)**			**UVA (CSS)**			**MVA (CSS)**		
	**HR**	**95% CI**	***P***	**HR**	**95% CI**	***P***	**HR**	**95% CI**	***P***	**HR**	**95% CI**	***P***
*Gender*												
Male	Ref						Ref					
Female	0.98	(0.76–1.28)	0.917				0.84	(0.60–1.17)	0.307			
*Age at diagnosis, years*												
≤ 65	Ref						Ref					
> 65	1.31	(1.02–1.68)	0.033	1.31	(1.01–1.69)	0.039	1.22	(0.90–1.65)	0.194			
*Race*												
White	Ref						Ref					
Black	1.10	(0.74–1.66)	0.619				1.19	(0.73–1.93)	0.468			
Asian/PI/AI	0.78	(0.52–1.19)	0.247				0.89	(0.55–1.44)	0.652			
*Tumor location*												
Upper	Ref						Ref					
Middle	0.96	(0.57–1.61)	0.901				0.95	(0.51–1.78)	0.892			
Lower	1.36	(0.84–2.21)	0.207				1.46	(0.82–2.59)	0.193			
*T category*												
T1-2	Ref						Ref					
T3-4	1.59	(0.96–2.64)	0.070				1.27	(0.89–1.82)	0.184			
*Lymph node metastasis*												
Negative	Ref						Ref					
Positive	0.93	(0.71–1.22)	0.621				1.01	(0.73–1.40)	0.914			
*Distant metastasis*												
No	Ref						Ref					
Yes	2.02	(1.49–2.74)	<0.001	2.04	(1.49–3.80)	<0.001	2.18	(1.50–3.17)	<0.001	2.17	(1.47–3.19)	<0.001
*Tumor size, mm*												
≤ 20	Ref						Ref					
> 20	1.59	(0.96–2.64)	0.070				1.85	(0.99–3.46)	0.052			
*Surgery*												
No	Ref						Ref					
Yes	0.51	(0.30–0.87)	0.014	0.65	(0.37–1.14)	0.135	0.50	(0.26–0.94)	0.034	0.64	(0.32–1.25)	0.196
*Radiation therapy*												
No	Ref						Ref					
Yes	0.54	(0.42–0.69)	<0.001	0.77	(0.59–1.01)	0.061	0.59	(0.43–0.80)	0.001	0.82	(0.59–1.14)	0.249
*Chemotherapy*												
No	Ref						Ref					
Yes	0.41	(0.31–0.54)	<0.001	0.40	(0.30–0.54)	0.001	0.49	(0.34–0.69)	<0.001	0.45	(0.31–0.65)	<0.001

CI: Confidence interval; CSS: Cancer specific survival; HR: Hazard ratio; MVA: Multivariate analysis; OS: Overall survival; UVA: Univariate analysis; SCEC: Small cell esophageal carcinoma.

**Figure 3.**
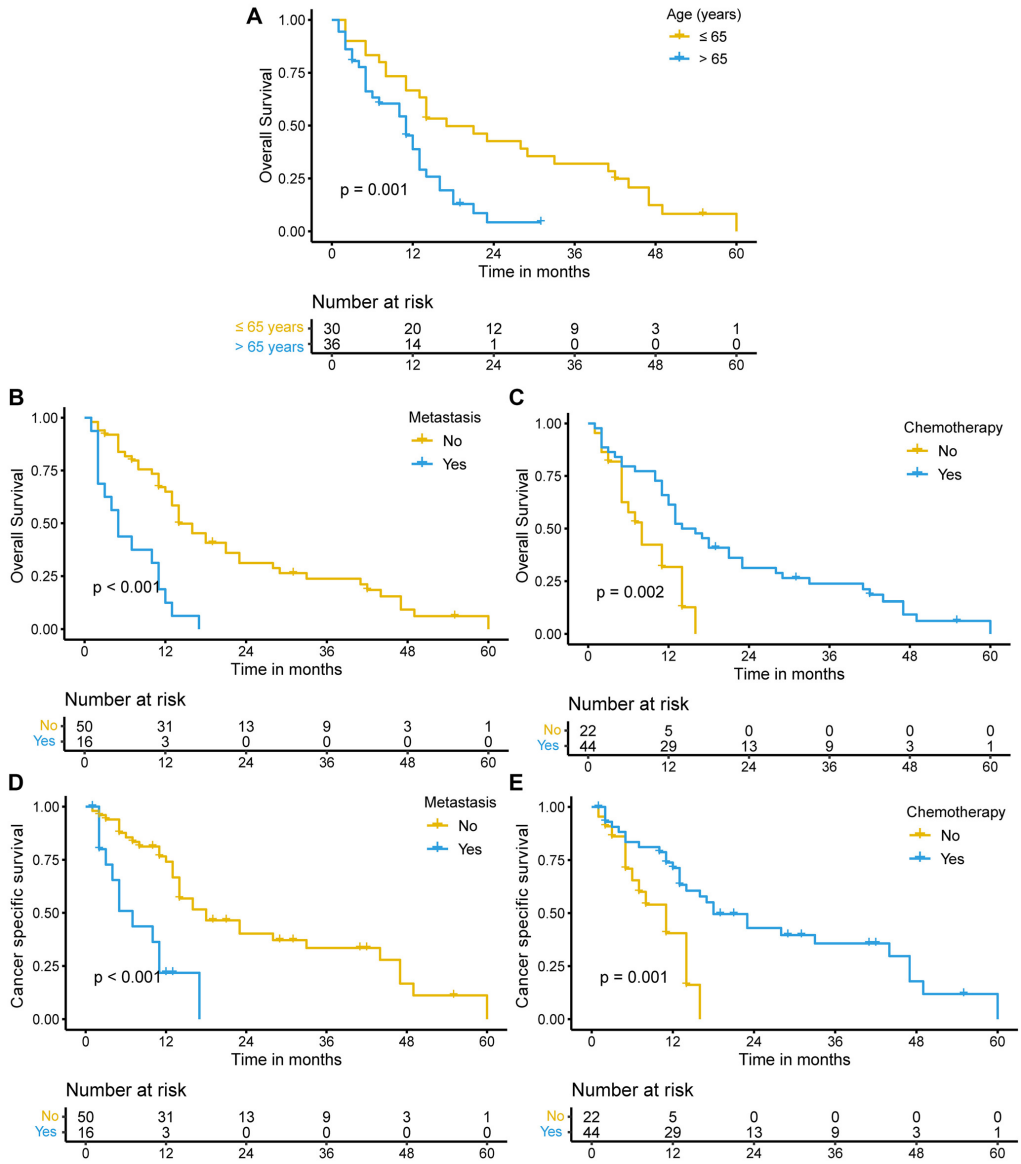
**Effect of age, distant metastasis, and chemotherapy on the prognosis of SCEC patients in the Chinese cohort.** Kaplan–Meier survival curves for OS in SCEC patients aged ≤ 65 years and > 65 years (A), with and without distant metastasis (B), and with and without chemotherapy (C). Kaplan–Meier survival curves for CSS in SCEC patients with and without distant metastasis (D), and with and without chemotherapy (E). SCEC: Small cell esophageal carcinoma; OS: Overall survival; CSS: Cancer-specific survival.

The independent prognostic factors identified from the multivariate Cox analysis for OS and CSS in the SEER cohort were also applied to the Chinese dataset. We observed similar findings in the Chinese cohort, with younger patients, no distant metastasis, and chemotherapy treatment associated with better OS (Figure 3A–3C) and CSS (Figure 3D and 3E). Additionally, we analyzed the therapeutic efficacy of different chemotherapy regimens, but no significant difference in OS (*P* ═ 0.55) or CSS (*P* ═ 0.46) was observed (Figure S2A and Figure S2B).

### Nomogram construction and validation

**Figure 4.**
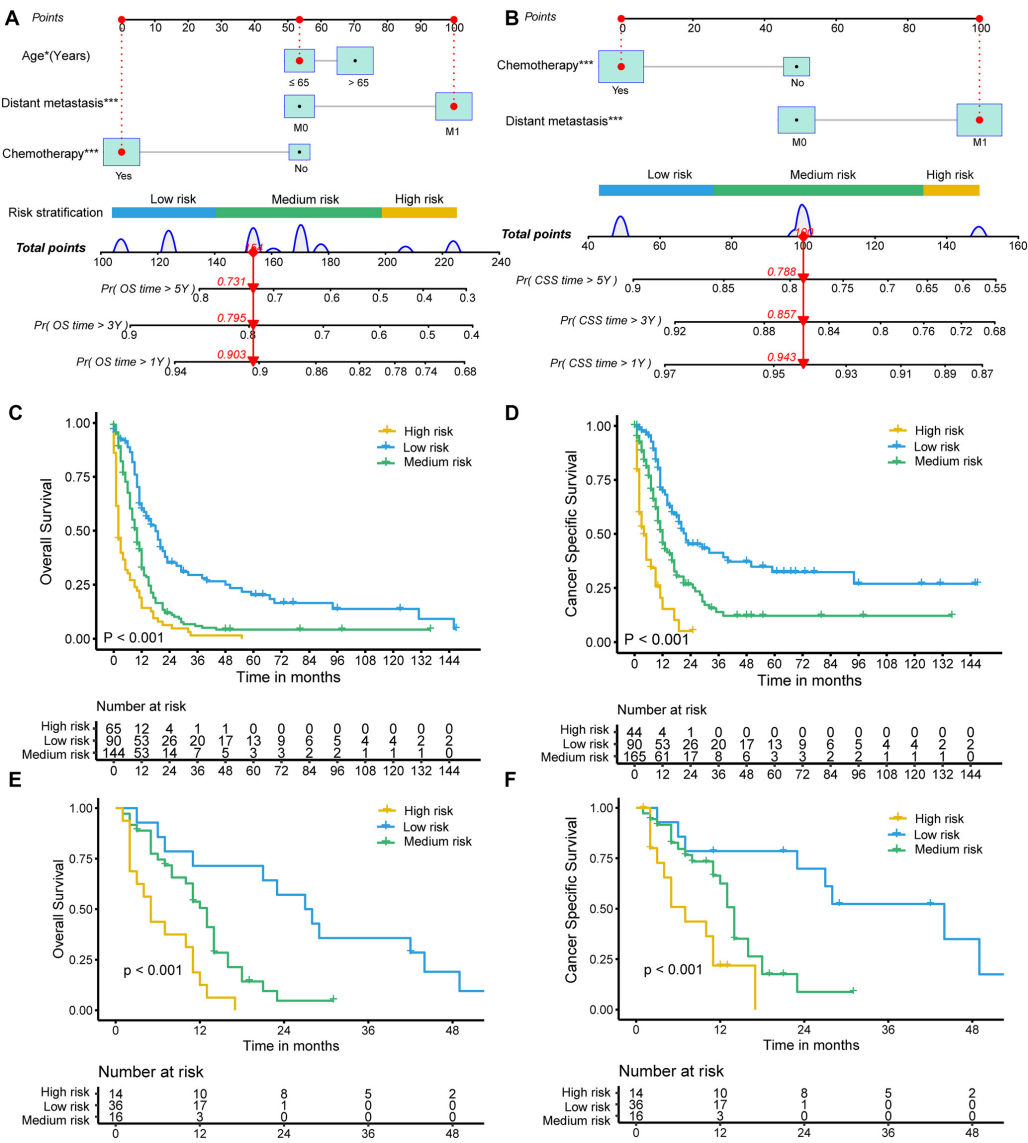
**Nomograms to predict OS and CSS for patients with SCEC and risk stratification.** Nomograms for OS (A) and CSS (B). Kaplan–Meier curves for OS (C) and CSS (D) in high-risk, medium-risk, and low-risk groups in the SEER cohort. Kaplan–Meier curves for OS (E) and CSS (F) in high-risk, medium-risk, and low-risk groups in the Chinese cohort. SCEC: Small cell esophageal carcinoma; OS: Overall survival; CSS: Cancer-specific survival; SEER: Surveillance, Epidemiology, and End Results.

The SEER SCEC cohort was used to establish nomograms for OS and CSS, with the Chinese dataset employed for validation. All independent factors identified in the prognostic analysis for OS and CSS were incorporated into the predictive models and presented as nomograms (Figure 4A and 4B). Using the nomogram, the risk score for each of the 299 SEER patients was calculated, and X-tile software was used to classify them into low-, medium-, and high-risk groups for OS and CSS prediction (Figure 4C and 4D). These risk groups demonstrated different survival outcomes. The high-risk group had a median OS of two months (95% CI 1–4) and a median CSS of four months (95% CI 2–9). The medium-risk group had a median OS of ten months (95% CI 8–11) and a median CSS of 12 months (95% CI 10–15). The low-risk group exhibited the longest median OS of 18 months (95% CI 12–22) and a median CSS of 22 months (95% CI 16–40). The 5-year OS rates varied among the risk groups: the high-risk group had not reached the 5-year OS, the medium-risk group had a 5-year OS rate of 4.2% (95% CI 1.8–9.8), and the low-risk group had a 5-year OS rate of 20.3% (95% CI 12.8–32.1). Similarly, the 5-year CSS rates differed: the high-risk group had not reached the 5-year CSS, the medium-risk group had a 5-year CSS rate of 12.2% (95% CI 6.6–22.7), and the low-risk group had a 5-year CSS rate of 32.3% (95% CI 22.2–47.2). Table 3 summarizes the median time, and the 1-year, 3-year, and 5-year rates for OS and CSS in the three risk groups. The nomograms applied to the Chinese dataset showed significant differences in OS (*P* < 0.001) and CSS (*P* < 0.001) among low-, medium-, and high-risk patients (Figure 4E and 4F). These findings were corroborated by the validation cohort.

**Figure 5.**
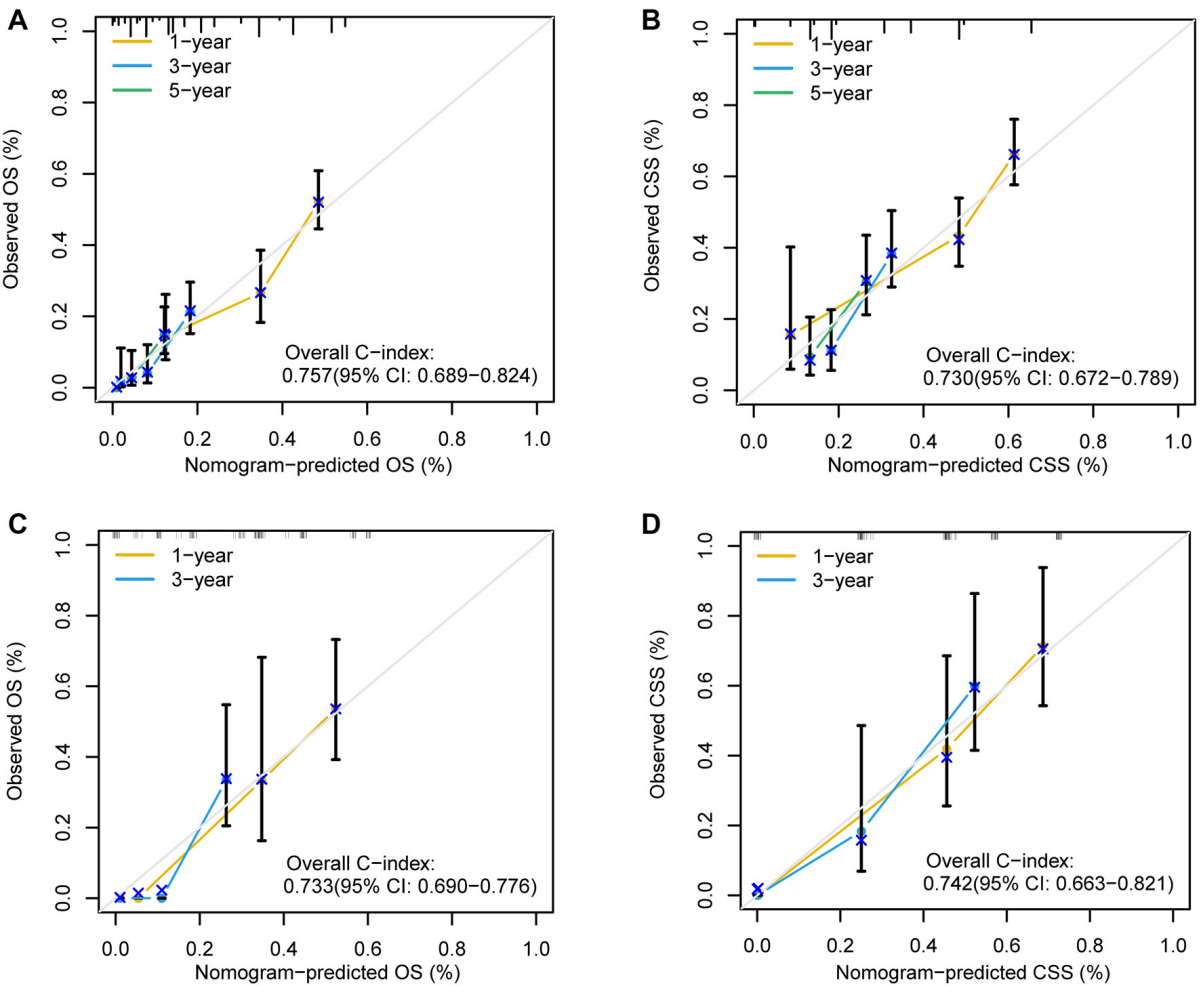
**Calibration curves of the nomogram of SCEC for 1-, 3-, and 5-year OS and CSS rates in the training cohort (A and B) and 1- and 3-year OS and CSS rates in validation cohort (C and D).** SCEC: Small cell esophageal carcinoma; OS: Overall survival; CSS: Cancer-specific survival.

**Figure 6.**
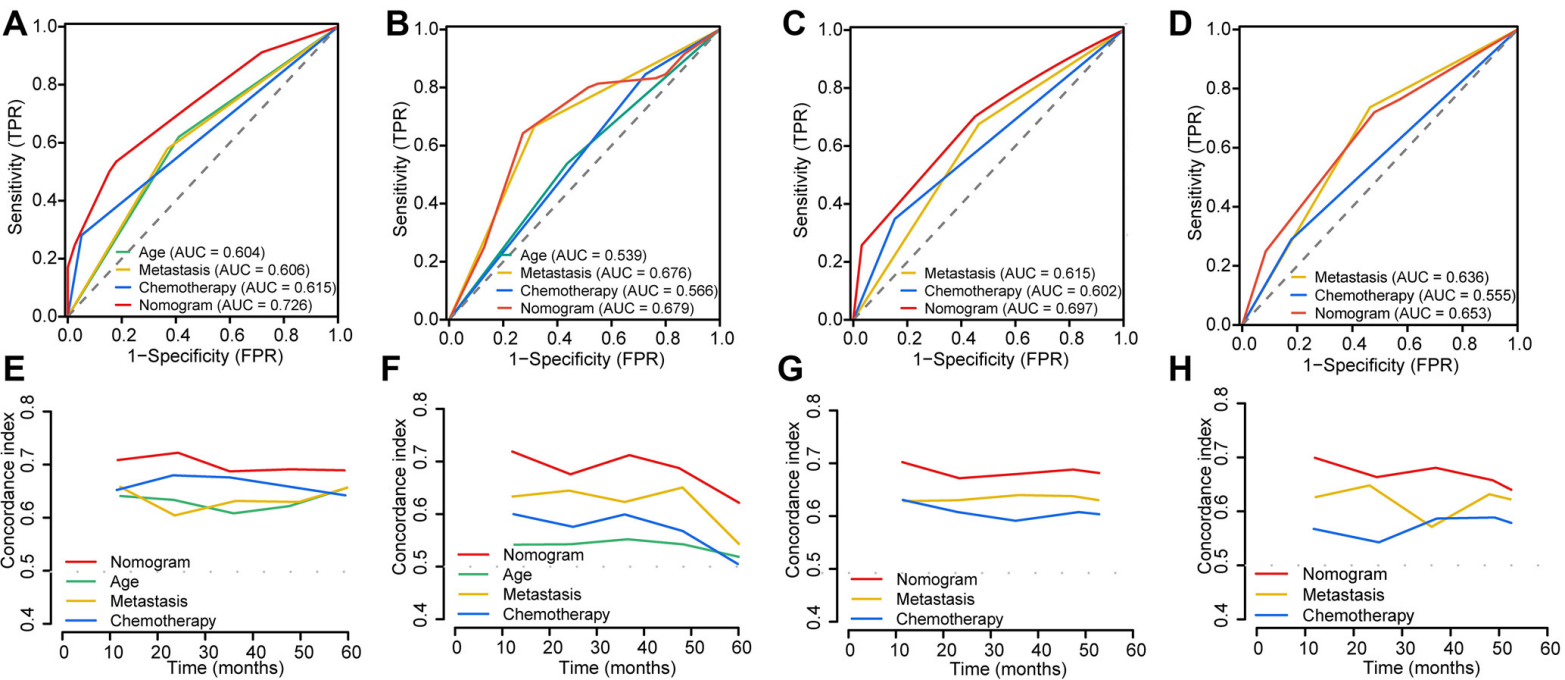
**Variable-dependent ROC curves of SCEC of OS and CSS in the training cohort (A and B) and validation cohort (C and D).** Time-dependent C-index curves for the nomogram of OS and CSS in the training cohort (E and F) and validation cohort (G and H). SCEC: Small cell esophageal carcinoma; OS: Overall survival; CSS: Cancer-specific survival; C-index: Concordance index; ROC: Receiver operating characteristic.

**Figure S1.**
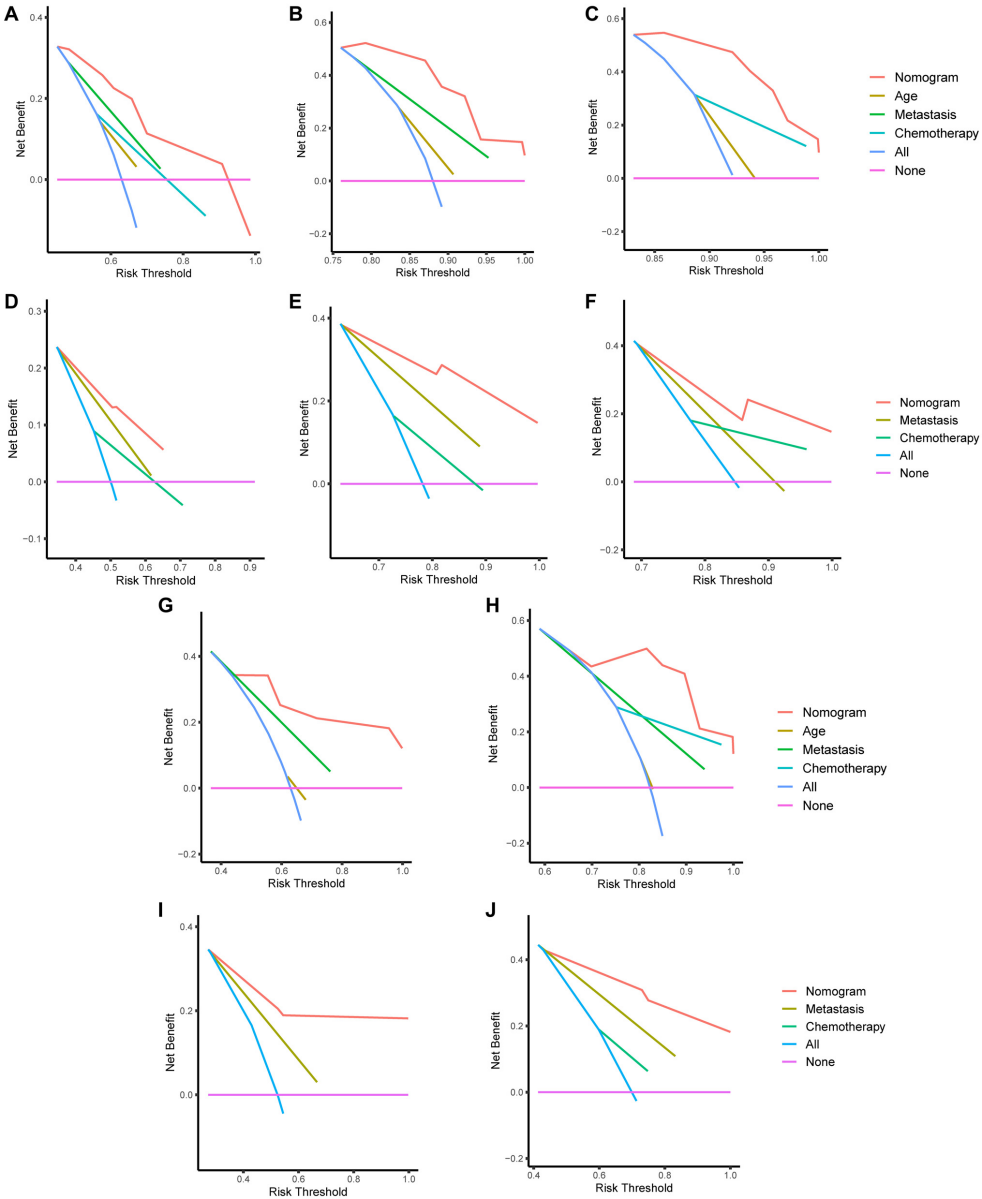
**Decision-curve analysis plots of the nomogram for small cell esophageal carcinoma (SCEC) predicting 1-, 3-, and 5-year overall survival (OS) rates (A--C) and cancer-specific survival (CSS) rates (D--F) in the training cohort.** Additionally, the decision-curve analysis plots of the nomogram for SCEC predicting 1- and 3-year OS rates (G--H) and CSS rates (I--J) in the validation cohort are shown. SCEC: Small cell esophageal carcinoma; OS: Overall survival; CSS: Cancer-specific survival.

The established nomograms were validated using several methods to assess their stability and efficacy. The calibration curves demonstrated strong agreement between actual observations and predicted probabilities for OS and CSS at one, three, and five years in both the training cohort (Figure 5A and 5B) and validation cohort (Figure 5C and 5D). The C-index values for the nomograms predicting OS and CSS in the SEER cohort were 0.757 (95% CI: 0.689–0.824) and 0.730 (95% CI: 0.672–0.789), respectively, indicating good discrimination. In the validation cohort, the nomogram for OS achieved a C-index of 0.733 (95% CI: 0.690–0.776), and the nomogram for CSS achieved a C-index of 0.742 (95% CI: 0.663–0.821), confirming the predictive performance of the nomograms in the validation cohort. Variable-dependent ROC analysis, incorporating age, metastasis, chemotherapy, and the nomogram, demonstrated superior predictive performance for OS in both the training cohort (Figure 6A) and validation cohort (Figure 6B). Similarly, the nomogram for CSS showed superior predictive value in both the training cohort (Figure 6C) and validation cohort (Figure 6D). Figure 6E and 6F displays the time-dependent C-index curves for OS in the training and validation cohorts, respectively. Figure 6G and 6H shows the time-dependent C-index curves for CSS in the training and validation cohorts. The DCA plots for the 1-, 3-, and 5-year rates of OS and CSS in both the training and validation cohorts are shown in Figure S1. These plots demonstrated that the nomogram for SCEC provided favorable net clinical benefits across a wide range of threshold probabilities, indicating high clinical utility.

**Table 3:** Risk stratification for the nomogram of OS and CSS in SCEC patients

**Risk group**		**Median time**	**1-year rate**	**3-year rate**	**5-year rate**
OS	High risk	2 months (95% CI 1–4)	14.3% (95% CI 7.8–26.2)	1.6% (95% CI 0.2–11.1)	NA
	Medium risk	10 months (95% CI 8–11)	32.5% (95% CI 25.4–41.4)	5.9% (95% CI 2.9–12.1)	4.2% (95% CI 1.8–9.8)
	Low risk	18 months (95% CI 12–22)	59.9% (95% CI 50.4–71.1)	29.5% (95% CI 20.9–41.6)	20.3% (95% CI 12.8–32.1)
CSS	High risk	4 months (95% CI 2–9)	15.4% (95% CI 5.9–40.2)	NA	NA
	Medium risk	12 months (95% CI 10–15)	45.4% (95% CI 37.6–54.9)	13.9% (95% CI 8.0–24.4)	12.2% (95% CI 6.6–22.7)
	Low risk	22 months (95% CI 16–40)	69.6% (95% CI 60.2–80.6)	41.3% (95% CI 30.9–55.1)	32.3% (95% CI 22.2–47.2)

OS: Overall survival; CSS: Cancer-specific survival; NA: Not available; SCEC: Small cell esophageal carcinoma.

## Discussion

SCEC is a rare neoplasm with aggressive behavior. SCEC patients generally have a poor prognosis, primarily due to the fact that most are diagnosed with metastatic disease at the time of diagnosis. However, the precise mechanisms underlying its development remain unclear. Evaluating the prognosis of SCEC presents challenges due to the limited available data on its occurrence and survival rates. In this study, we aimed to address this gap by investigating the frequency of SCEC and analyzing the survival outcomes of patients using both the SEER database and an external real-world cohort. Furthermore, we developed prognostic nomograms to facilitate the prediction of OS and CSS in SCEC patients.

In the current SEER cohort study, we found that distant metastasis was an independent prognostic factor for both OS and CSS, consistent with previous findings [2, 8]. Approximately half of SCEC patients experience distant metastasis at the time of diagnosis, greatly diminishing their survival prognosis. Among these metastasis sites, the liver (21.4%) is the most common, followed by the lungs (6.7%), bones (6.4%), and the brain (1.3%). This pattern of metastatic occurrence was similar to the Chinese data in this study and in other retrospective studies [2]. Age was also identified as a significant prognostic factor in SCEC patients, with those under 65 exhibiting better OS and CSS compared to those aged 65 or older. Dysphagia is typically the initial symptom observed in patients with esophageal carcinoma, and it is often accompanied by malnutrition. Nutrient depletion can result in reduced tolerance to chemotherapy and a compromised immune system, accelerating tumor progression and diminishing survival outcomes [9]. While tumor location was not identified as a prognostic factor, distinct distribution characteristics were observed in this study. Most tumors were found in the mid- and lower esophagus, aligning with the findings of two retrospective studies conducted in China [2, 10]. This could be due to the higher abundance of Merkel cells in the middle section of the esophagus and the presence of endocrine cells in the cardiac glands located in the distal part of the esophagus [11]. All previous reports on SCEC, including this study, have found that the proportion of male patients is significantly higher than that of female patients [2, 8, 10, 11]. A recent study by Wang et al. [12] explored potential risk factors for esophageal neuroendocrine neoplasms, identifying alcohol consumption and cigarette smoking as significant risk factors. Notably, individuals who engage in both habits exhibit the highest susceptibility to developing esophageal neuroendocrine neoplasms. Another study observed that a heavy smoking history serves as a risk factor for SCEC development [13].

The optimal treatment for non-metastatic SCEC remains uncertain, despite the common use of surgery combined with neoadjuvant or adjuvant chemotherapy, as well as definitive chemoradiotherapy. The European Society for Medical Oncology (ESMO) guidelines suggest platinum-based doublet therapy for managing gastroenteropancreatic neuroendocrine neoplasms (GEP-NENs) [14], while the National Comprehensive Cancer Network (NCCN) guidelines recommend a combination of chemotherapy and radiotherapy [15]. A large-scale retrospective study demonstrated that locally advanced SCEC patients treated with chemotherapy alone had worse survival compared to those who underwent chemoradiotherapy [16]. Another study from Japan also showed favorable treatment efficacy with chemoradiotherapy [17]. Therefore, definitive chemoradiotherapy may be a promising therapeutic approach for locally advanced SCEC, warranting further investigation. For patients with metastatic or recurrent SCEC, the optimal first-line chemotherapy regimen remains unclear. Two commonly used regimens, based on small cell lung cancer (SCLC) treatment, etoposide plus cisplatin (EP) and irinotecan plus cisplatin (IP), are recommended for GEP-NENs by NCCN guidelines. However, there is no clear evidence to determine the superior choice [15]. Our study also compared the therapeutic efficacy between these two chemotherapy regimens, but no significant difference in OS and CSS was observed. This finding is consistent with the results of the TOPIC-NEC study, a phase 3 randomized clinical trial comparing OS outcomes between the EP and IP regimens in patients with advanced NEC of the digestive system [18]. The trial enrolled 170 patients, including 15.5% of esophageal NEC patients in the EP group and 9.3% in the IP group. Subgroup analysis did not reveal significant differences between the two groups. The trial suggested that both EP and IP regimens could be considered as first-line chemotherapy options for advanced gastrointestinal NEC. In summary, chemotherapy plays a crucial role in treating SCEC, which aligns with our study findings. Besides the regimen, the course and dose of chemotherapy also matter. A study by Jeene et al. demonstrated that patients achieved the best outcomes when receiving at least four cycles of chemotherapy [19]. Another study observed that an insufficient total chemotherapy dose, resulting from dose adjustments, compromises treatment efficacy [2].

With the emergence of immunotherapy and the encouraging results from the CASPIAN and IMpower133 trials, regimens combining chemotherapy with anti-programmed death ligand 1 (PD-L1) antibodies might also be effective in primary extrapulmonary small cell carcinoma (ESCC) treatment [20, 21]. Salhab et al. [22] found a trend toward higher response rates to standard chemotherapy with or without radiotherapy, as well as improved survival, among ESCC patients with PD-L1 positivity. A recent study by Yamashita et al. demonstrated that a significant proportion of SCEC cases exhibited PD-L1 combined positive scores (CPS) of ≥1 and ≥10 (60% and 33%, respectively), indicating the potential of PD-L1 as a therapeutic target for this highly aggressive neoplasm. Additionally, the study observed a correlation between PD-L1 expression and high levels of tumor-infiltrating lymphocytes (TILs) [23]. Another study on PD-L1 expression in the digestive system NEC found that 67% (2/3) of SCEC cases exhibited positivity in tumor cells or tumor-associated immune cells, similar to squamous-cell carcinoma [24]. This suggests that PD-1/PD-L1 therapy may be a promising treatment strategy for SCEC. Two recent case reports demonstrated the remarkable effectiveness of combining anti-PD1 immunotherapy with anti-angiogenic therapy. Camrelizumab (a PD1 inhibitor) or tislelizumab (a PD1 inhibitor) combined with anlotinib (a multitarget tyrosine kinase inhibitor) were administered as two or more lines of therapy for advanced esophageal NEC [25, 26]. Both patients achieved more than 20 months of survival. Further studies with larger sample sizes are needed to validate its efficacy.

Several recent studies have developed nomograms for predicting SCEC prognosis [27–30]. Although the variables used to construct the nomograms varied, all four studies included age and chemotherapy, similar to our study. The populations in the studies by Li et al. [27] and Qie et al. [28] were from the SEER database, with time periods from 1973–2015 and 1975–2016, respectively. Internal validation was performed after constructing nomograms. Although expanding the time period can include more participants, the heterogeneity caused by changes in diagnostic criteria and missing values could affect the reliability of the results. Our study design is similar to that of Liu et al. [29], which used SEER data for model building and in-house data for validation. However, we found that Liu et al.’s study population was too broad. Based on our analysis, we believe that primary tumor indicators may not have been considered in Liu et al.’s study, potentially including SCEC cases with other primary cancers. Since multiple tumors significantly impact survival prognosis, we believe the inclusion criteria in Liu et al.’s study were not stringent enough, which may have affected the reliability of the results. The study by Zhang et al. [30] was a single-center retrospective study that performed only internal validation, lacking broader applicability. Additionally, the study did not specify clear patient inclusion and exclusion criteria. In summary, our study extended the inclusion time interval using strict criteria, ensuring that the cases included are of higher quality. Additionally, we used multi-center retrospective data for model construction and external validation, making our results more reliable and universally applicable.

However, our study has several limitations. Firstly, the number of cases is relatively small compared to other commonly studied histologies. Secondly, the SEER database lacks complete information on variables such as surgical modality and chemoradiotherapy regimen, which could affect the predictive power of our model. Thirdly, there is the potential for misdiagnosis among different medical centers due to standardized diagnostic criteria for SCEC, and the pathological information cannot be verified in the SEER database. Lastly, selection biases were inevitable in this retrospective analysis.

## Conclusion

SCEC is a rare and aggressive tumor with poor survival outcomes. Chemotherapy plays a crucial role in the treatment of SCEC and is an independent factor, along with distant metastasis. Further prospective clinical trials are essential to investigate and validate more effective therapeutic strategies.

## Supplemental data

**Table S1:** ICD-O-3 morphology codes for identification of cases

**Small cell carcinoma**	**Site codes**
8041/3: Small cell carcinoma NOS (295)	C15.0 Cervical esophagus C15.1 Thoracic esophagus
8042/3: Oat cell carcinoma (4)	C15.2 Abdominal esophagus C15.3 Upper third of esophagus
8043/3: Small cell carcinoma, fusiform cell (0)	C15.4 Middle third of esophagus C15.5 Lower third of esophagus
8045/3: Combined small cell carcinoma (0)	C15.8 Overlapping lesion of esophagus C15.9 Esophagus, NOS

**Figure S2.**
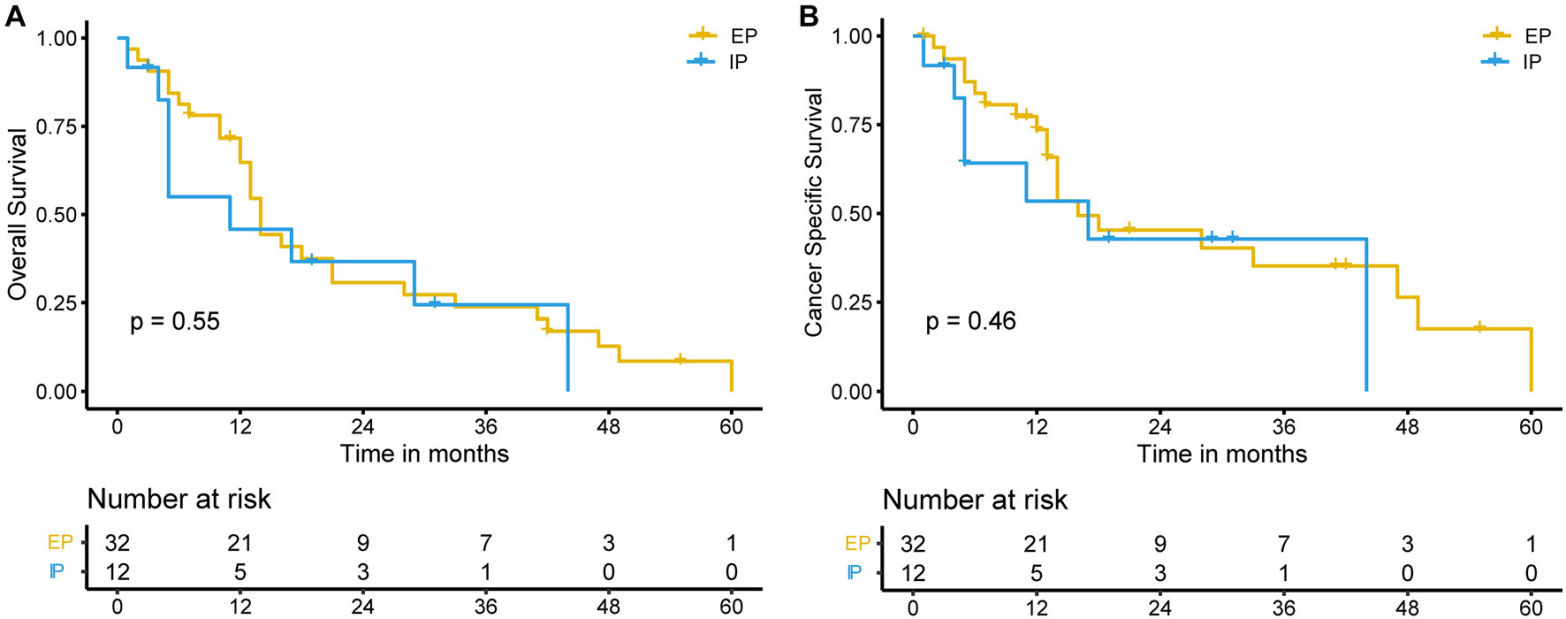
**Effect of chemotherapy regimen on the prognosis of SCEC patients in the Chinese cohort.** Kaplan-Meier survival curves for overall survival (OS) (A) and cancer-specific survival (CSS) (B) in SCEC patients treated with the EP or IP regimen. EP: Etoposide and cisplatin; IP: Irinotecan and cisplatin.

## Data Availability

The original contributions presented in the study are included in the article/supplementary material. Further inquiries can be directed to the corresponding author.
